# Analysis of Risk Factors and Association of Cluster of Differentiation (CD) Markers With Conventional Markers in Delayed Fracture Related Infection for Closed Fracture

**DOI:** 10.7759/cureus.20124

**Published:** 2021-12-03

**Authors:** Archana Raikwar, Ajai Singh, Vikas Verma, Abbas Ali Mehdi, Narendra Singh Kushwaha, Rashmi Kushwaha

**Affiliations:** 1 Paediatric Orthopedics, King George's Medical University, Lucknow, IND; 2 Biochemistry, King George's Medical University, Lucknow, IND; 3 Orthopedic Surgery, King George's Medical University, Lucknow, IND; 4 Heamatopathology, King George's Medical University, Lucknow, IND

**Keywords:** serum inflammatory markers, risk factors, cd66b, cd64, fracture related infections

## Abstract

Background: Fracture-related infections (FRI) remain a difficult consequence for orthopedic trauma patients, their relatives, the treating physicians, and the healthcare systems. Delayed fracture-related infection is an important step in the infection process that can be controlled by diagnosing and preventing it from moving to the next level. Neutrophils CD64 and CD66b were identified as sensitive indicators in the event of infection. Normal sequential changes, on the other hand, occur after surgery and are extremely high. They are back to normal on the 10^th^ day after the operation. The aim of this study was, therefore, to examine the risk factors associated with fracture-related infection by comparing cluster of differentiation (CD) indicators with conventional markers and comparing them with gold standards culture reports. As a result, it could be an early sign of a closed fracture infection.

Material & Methods: Between February 2020 and March 2021, 510 patients from the Department of Orthopedics at King George Medical University in Lucknow agreed to participate in the study. The study included patients who had a closed fracture and had undergone elective or emergency surgery. Blood was withdrawn before the surgery (baseline) on day one and again on the third, seventh, and 10^th^ day after the operation to measure the quantitative measurements of the biomarkers (total leucocyte count [TLC], erythrocyte sedimentation rate [ESR], C reactive protein [CRP], CD64, and CD66b) in all follow-up examinations. Patients were monitored for delayed signs of the infection for 2 to 10 weeks. The biomarkers were evaluated and linked to the culture reports.

Results: Of the 510 patients included, 272 were men (53.3%) and 238 women (46.7%), the mean age was 40 (20-78), the mean age for fracture related infection with positive culture (FRI POS) was 48.0 (SD: 19.47), for fracture related infection with negative culture (FRI NEG) was 46.20 (SD: 17.18), and for patient with no signs of infections (NON-FRI) was 45.13 (SD: 17.62) (p <0.001), the mean duration of the fracture to admission (in hours) was 4.90 (SD: 1.92), 4.91 (SD: 2.65), and 5.14 (SD: 2.66) (p <0.001), respectively. The mean duration of admission to surgery (in hours) was 31.54 (SD: 85.14), 43.14 (SD: 105.64), and 61.84 (134.14), respectively (p <0.001). The mean duration of surgery was 4.63 (SD: 1.85), 5.14 (SD: 2.16), and 5.05 (SD: 2.16) (p <0.001). The risk factors such as bone type (p = 0.04) and addiction (p = 0.01) were identified as statistically significant. There was no correlation between the CD66b markers on the third, seventh, and 10^th^ days. CD64 was significantly correlated with ESR, TLC, and CRP on the 10^th^ day in the FRI-positive group (r = 0.638; p = 0.03) (r = 0.744; p = 0.009) (r = 0.817; p = 0.002).

Conclusion: The risk factors for infection in fracture patients are significantly influenced by the type of bone and addiction the patient is using. Elevated CD64 levels could be used as a diagnostic marker for infection early on the 10^th^ day after surgery before the appearance of clinical signs.

## Introduction

Fracture-related infection (FRI) is still a difficult complication for orthopedic trauma patients, their families, and treating physicians, as well as healthcare systems [1]. Acute, chronic, early, delayed, and late infections are among the various classifications that separate FRI into discrete groups. We are primarily interested in delayed fracture-related infections in this investigation. Delayed infections occur after two weeks and within 10 weeks and are usually caused by less aggressive bacteria like *Staphylococcus Aureus and Acinetobacter calcoaceticus.* As the illness progresses, biofilms mature and grow more resistant to antibiotic therapy and human defense. The delayed stage of infection is vital to retain the perpetuity of the early infection because, at this stage, the infection might be regarded or saved rather than becoming worse [2]. The pathophysiology of an infection following a closed fracture is a topic of study. First, it appears that bacteria can infect healthy tissue and body fluids and that an open wound is not the primary source of bacterial infection. Second, mechanisms that allow such indwelling bacteria to ‘‘home" to closed injury sites have been described. Third, it has been proposed that changes in the local environment following a closed injury can enhance infection susceptibility. Infection at a closed fracture site is frequently misdiagnosed. After several weeks of immobilisation, it is plausible to suspect infection in individuals who continue to have pain and edema at the fracture site [3]. To diagnose infections, serum inflammatory indicators such as leukocyte count, C-reactive protein, and erythrocyte sedimentation rate have been tested. All of them are non-specific and can be elevated after a trauma or in a variety of different inflammatory disorders [4]. Neutrophil CD64 (nCD64) expression appears to be a potential marker of bacterial infections, which has been brought into action to fill in the gaps and confusions. CD64 expression is a useful diagnostic for detecting sepsis in severely unwell individuals early on. CD64 is a high-affinity immunoglobulin Fc receptor that is expressed continuously on monocytes and eosinophils. Much research has been conducted to identify the role of neutrophil CD64 (nCD64) expression in the diagnosis of bacterial infection and sepsis in recent years [5]. As a result, CD64 may be an infection-specific marker, as it has been proven to be useful in detecting both systemic and local infections. CD66b, on the other hand, belongs to the immunoglobulin-like subfamily of carcino-embryonal antigens and is a glycosylphosphatidylinositol-anchored membrane protein. It's found in the membranes of particular and gelatinase-containing neutrophil granules and triggers like N-Formylmethionyl-leucyl-phenylalanine (fMLP) cause it to migrate to the cell surface [6]. CD66b marker was discovered to be an efficient biomarker for the prediction of early bacterial infection when used in combination with peripheral blood CD66b+CD10 [7]. As a result, we chose to investigate the marker's diagnostic capabilities individually.

## Materials and methods

The study was a single-center observational study and was approved by King George's Medical University U.P. Institutional Ethical Committee with approval 100^th^ ECMIIBPHD./P4. We retrospectively reviewed 510 patients who underwent elective or emergency surgery for closed fracture at an orthopedic trauma from April 2020 to March 2021. The population included patients who had closed fractures by any means and had undergone elective or emergency surgery. Their blood samples were taken before surgery and were taken again on the third, seventh, and 10^th^ Day after the surgery to look for their elevations, and the same were correlated with the conventional markers (C reactive protein [CRP], erythrocyte sedimentation rate [ESR], total leucocyte count [TLC]) with that of test markers (CD64 and CD66b). All patients more than 18 years of age with a closed fracture, who had undergone elective or emergency surgery, were included in the study. Cases below 18 years, cases with any type of infections, or cases where any type of medications was taken were excluded from the study. As per Metsemakers et al., to look for any type of infection, the criteria are between 2 weeks to 10 weeks (for delayed infections). Patients were observed for 2 weeks to 10 weeks for confirmatory signs for infections (fistula, sinus, wound breakdown, purulent drainage, or presence of pus) or suggestive signs of infection (redness and fever elevated serum markers). Based on these symptoms and culture reports, patients were divided into three groups as fracture-related infection with positive culture (FRI POS), fracture-related infection with negative culture (FRI NEG), patient with no signs of infections (NON-FRI). Patient’s demographical data such as age, sex, education, occupation, and fracture-related data such as the reason for fracture, Tscherne classification for closed fracture, addiction, comorbidity was analyzed to find the risk factor associated with infection. The outcome measure included the risk factors associated with infection and correlation of cluster of differentiation (CD) markers with conventional markers at baseline and follow-ups. Chi-squared tests were used to compare categorical data. The paired t-test was used to compare markers from baseline to follow-up. Pearson’s Correlation was used to see the correlation between the CD markers and conventional markers. Statistical analysis was done by IBM Corp. Released 2013. IBM SPSS Statistics for Windows, Version 22.0. Armonk, NY: IBM Corp.

## Results

Out of 510 patients enrolled, 272 were males (53.3%), and 238 were females (46.7%); the mean age was 40 (20-78). Of the patients enrolled 138 (27.1%) had primary education, 83 (16.3%) had secondary, 59 (11.6%) were intermediate, 86 (16.9%) were graduate, 24 (4.7%) were postgraduate 85 (16.7%) could sign, and 35 (6.9%) were illiterate. As per the occupation of patients, 41 (8.0%) were in service, 120 (23.5%) were farmers, 87 (17.1%) were housewives, 126 (24.7%) were students, 54 (10.6%) were business persons, and 82 (16.1%) were laborers. The most affected bone was humerus 119 (23.3%), followed by tibia 102 (20.0%), ulna 82 (16.1%), radius 80 (15.7%), fibula 67 (13.1%), and femur 60 (11.8%). As per the Tscherne classification for closed fracture, 44 (8.6%) were of grade 0, 143 (28.0%) were of grade 2, 215 (42.2%) were of grade 3, and 108 (21.2%) were of grade 4. The mean duration of fracture to admission was 6.06 (2-15) in hours. The mean duration of admission to surgery was 55.36 hours (2-504), and the mean duration of surgery was 5.24 hours (2-9). Two-hundred and seventeen (42.5%) cases were of road accidents, while 77 were (15.1%) due to injury caused by fights, 82 (16.1) cases were of people who fell from a height, 78 (15.3%) were due to slip injury, and 56 (11.0%) had other reasons like fights among themselves, machine injuries, and cattle attacks. The surgery was either elective 268 (52.5%) or emergency 242 (47.5%). Some of the patients were suffering from comorbidity 144 (28.2%), like hypertension 39 (27.08%), diabetes 105 (72.91), etc. Among the patients, 226 (44.3%) had an addiction to smoking, while 99 (19.4%) patients consumed alcohol and were also smokers (Table 1).

**Table 1:** Association of Factors with Fracture Related Infections NON FRI: non fracture related infection, FRI POS: fracture related infection positive, FRI Negative: fracture related infection negative

Parameters	NON FRI(465)	FRI POS(11)	FRI NEG (34)	Chi	P value
Gender					
Male	245	6	21	1.05	0.59
Female	220	5	13		
Education of Patient				8.96	0.70
Primary	124	4	10		
Secondary	77	2	4		
Intermediate	51	1	7		
Graduate	81	0	5		
PG	22	1	1		
Can sign	80	2	3		
Illiterate	30	1	4		
Occupation of patient					
Service	39	0	2	5.09	0.88
Farmer	107	2	11		
Housewife	81	1	5		
Student	113	4	9		
Business	50	2	2		
Labourer	75	2	5		
Reason for Fracture					
Road accident	197	4	16	10.69	0.22
Fighting injury	72	3	2		
Fall from height	79	1	2		
Slip	70	1	7		
Others	47	2	7		
Type of Surgery					
Elective	249	5	14	2.17	0.33
Emergency	216	6	20		
Type of Bone					
Radius	71	0	9	18.38	0.04*
Humerus	111	2	6		
Ulna	77	1	4		
Tibia	91	2	9		
Fibula	62	1	4		
Femur	53	5	2		
Long Bone					
Type of Surgery					
Proximal	103	3	9	1.04	0.90
Distal	232	6	15		
Diaphyseal	130	2	10		
Comorbidity					
Yes	136	4	4	5.14	0.07
No	329	7	30		
Addiction					
Smoking	211	5	10	12.79	0.01*
Both	92	4	3		
No	162	2	21		
T-Scherne Classification					
Grade 0	40	3	1	10.69	0.09
Grade 1	136	2	5		
Grade 2	192	4	19		
Grade 3	97	2	9		

The mean age for FRI POS was 48.0 (SD: 19.47), for FRI NEG was 46.20 (SD:17.18), and for NON FRI was 45.13 (SD: 17.62) (p<0.001). The mean duration of fracture to admission (in hours) was 4.90 (SD:1.92), 4.91 (SD: 2.65), 5.14 (SD: 2.66) (p<0.001). The mean duration of admission to surgery (in hours) was 31.54 (SD:85.14), 43.14 (SD:105.64), and 61.84 (134.14) (p<0.001). The mean duration of surgery (in hours) was 4.63 (SD: 1.85), 5.14 (SD: 2.16), and 5.05 (SD: 2.16) (p<0.001), respectively (Table 2).

**Table 2:** Comparison of continuous variables in patients with FRI POS, FRI NEG, and NON FRI NON FRI: non fracture related infection, FRI POS: fracture related infection positive, FRI NEG: fracture related infection negative

Variables	FRI POS	FRI NEG	NON FRI	P Value
Age	48.0 (19.47)	46.20 (17.18)	45.13 (17.62)	0.000*
Duration of fracture to admission (in hours)	4.90 (1.92)	4.91 (2.65)	5.14 (2.66)	0.000*
Duration of admission to surgery (in hours)	31.54 (85.14)	43.14 (105.64)	61.84 (134.14)	0.000*
Duration of surgery (in hours)	4.63 (1.85)	5.14 (2.16)	5.05 (2.16)	0.000*

Correlation between CD64 and CD66b with ESR TLC and CRP

CD66b and TLC were significantly negatively correlated in the patient group FRI Positive (r= -0.628; p=0.03); however nothing significant could be detected in other groups at baseline. When correlated at 10^th^ day CD64 & ESR, TLC and CRP were significantly correlated in FRI positive group (r=0.638; p=0.03) (r=0.744; p=0.009) (r=0.817; p=0.002), respectively.

In the FRI POS group, the values of CD64 measured on baseline (before surgery) were elevated at day three (8700 (5678-8976) mol/cell) and peaked at day seven (10890 (9078-10892) mol/cell), and slightly elevated at day 10 (10326 (9825-13787) mol/cell). The values of CD66b measured at baseline (456 (345-678) mol/cell) were slightly elevated at day three, 824 (568-981), and highly elevated at day seven (7658 (5679-8788) mol/cell), and slightly lower at day 10 (6750 (5642-9780) mol/cell). All the values were significantly correlated (Table 3).

**Table 3:** Serial values of CD64 and CD66b, TLC, ESR, and CRP (median (interquartile range 25/75) at baseline and at follow up (third, seventh, and 10th day among the groups FRI positive, FRI negative and NON FRI) CRP: C reactive protein, ESR: erythrocyte sedimentation rate, TLC: total leucocyte count, CD: cluster of differentiation

Culture	Parameter	Baseline	Third Day	Seventh Day	10^th^ Day
FRI POS	ESR	6 (4-9)	22* (14-36)	14* (12-69)	20* (16-72)
	TLC	6700 (4562-8798)	8534* (6780-11500)	8200* (6746-16000)	17000** (16555-19457)
	CRP	2.0 (1-2)	7.89* (5.2-9.6)	25.67** (22.46-28.9)	5.23* (2.1-8.58)
	CD64	3456 (2467-5678)	8700* (5678-8976)	10890** (9078-10890)	10326** (9825-13787)
	CD66b	456 (345-678)	824* (568-981)	7658** (5679-8788)	6750** (5642-9780)
FRI NEG	ESR	6 (4-7.25)	18* (7-34)	14.5* (10-19)	16* (12-22)
	TLC	6984 (6625-7843)	8000 * (6675-9819)	8000 * (5919-9987)	7890* (6650-9850)
	CRP	2.32 (1.9-2.72)	8.27** (5.36-16.02)	21.62** (15.1-25.64)	3.43* (2.88-4.71)
	CD64	3426 (2331-6810)	7776** (5625-9165)	7233** (6563-9876)	6571** (5539-8980)
	CD66b	371 (321-678)	727** (672-878)	495 (324-657)	413 (276-671)
NON FRI	ESR	6* (5-8)	22* (11-44)	16* (12-46)	20* (14-56)
	TLC	7767 (6542-8755)	8415** (6700-11555)	8600** (6800-5300)	8000** (6724-9655)
	CRP	2.13 (1.90-2.87)	8.65** (5.15-15.6)	22.54** (15.67-29.24)	4.13** (2.98-7.39)
	CD64	3433 (2265-7517)	7834** (5635-9824)	8567** (6727-9397)	4567 (3467-5673)
	CD66b	460 (345-672)	654** (456-789)	543** (350-780)	324** (253-386)

## Discussion

There is no sufficient study on an infection associated with closed fracture. The results obtained from the study are worth observing. CD64 are elevated after the surgery; however, the infection should be diagnosed as early as possible. We tried to diagnose the infection as early as possible (10^th^ day) with a culture test. We observed that the elevations occurred in all patients undergoing surgery, and it began to subside after the seventh day, and it came to normal on the 10^th^ day. Our study showed high elevations in CD64 and CD66b markers and was statistically significant.

The diagnosis of musculoskeletal infection can be difficult since hematological investigations are not reliable, and a negative bacterial culture does not necessarily exclude infection. In addition, the CRP and ESR can increase in the presence of an inflammatory process even in the absence of infection [8]. There have been many reports on the efficacy of CD64 as a diagnostic marker for infection in patients with rheumatoid arthritis (RA) and those in the early postoperative period and musculoskeletal infections, but the expression of the marker and sequential changes at days after the surgery has never been studied in fracture-related infections. Therefore, we measured the expression of CD64 and CD66b along with conventional markers TLC, ESR, CRP before the surgery and at the third, seventh, and 10^th^ day after the surgery. Expressions were increased on the third day and peaked on the seventh day, and returned to the baseline, or there was a decrease in patients with no infections but elevated on the 10^th^ day in patients with infections. However, on the 10^th^ day, we found a statistically significant difference in the expression of CD64 [9]. A similar study conducted by Narutaka Katoh et al. in 2013 on total hip joint arthroplasty infection showed the levels of CD64 significantly elevated from day one, peaked at day three, and decreased significantly after day five. Statistical analysis confirmed that significant differences existed between the baseline level and the levels at days one and three, while no significant differences existed between the baseline level and those at days five, seven, or 14, and concluded that the CD64 levels rise significantly, peaking within about three days following normal total joint arthroplasty but decreases rapidly to near baseline within five days. The data obtained can be expected to form a possible basis for early diagnosis of postoperative periprosthetic infection, of which, in our study, the maximum rise in CD64 was at day seventh and day 10^th^ [10].

As per the 2019 study of Gi Ho Moon et al., the risk factors for infection in fracture patients were found to be significantly influenced by open fracture rather than the underlying disease or anatomical feature of the patient as compared to other studies as [11] in our study the risk factors were also the cause for infection in closed fracture and not any other reason.

The main drawback for conducting studies related to fracture-related infection is that no clear definition is there in the study conducted by M Morgenstern in 2018 [12], which included a questionnaire related to the actual definition. It was found that 90% of respondents agreed with the consensus definition. “Positive culture”, purulent drainage”, and increased CRP were considered important symptoms for infections, and it was concluded that such definition will enable a clinician to standardize future research as well as improve the quality of diagnostics tools and treatment algorithms.

There are many studies in the pipeline which are trying to find different ways to improve the diagnosis and treatment. Melissa Depypere et al. [13], in a review, stated that the available evidence in FRI is scarce, and the available evidence's primary focus is on prosthetic joint infections (PJI). This study provides recommendations for systemic antimicrobial therapy concerning FRI. However, she reported that there is an urgent need to standardize recommendations and antimicrobial therapies.

One of the major risk factors is fall-related injury. It is an important public health issue among older adults as they are one of the leading causes of fall-related injury and death in the population. Several studies have identified the characteristics and potential risk factors for fall-related fractures. However, there are limited studies on risk factors and preventive measurements for in-hospital complications of fall-related fractures [14]. In our study, the rate of fall injury was not found significant.

Patients with infection had considerably greater rates of hospital readmissions, emergency department visits, and healthcare expenses than patients without infection, according to a new study. This real-world study revealed a two-year increase in infection rates, as well as a considerable increase in healthcare resource utilization and expenses. However, we did not include the cost, but our institution is currently working on a paper that will include several other factors [15].

Our study suggests that CD64 and CD66b are helpful markers in the diagnosis of fracture-related infection as early as possible. The clinical symptoms occur after two weeks of surgery, and the infection keeps on developing, which is quite late. Hence, increased levels of biomarkers on the 10^th^ day from the surgery could be considered as the initiation of infection at the fracture surgery site. Without a doubt, the culture is the gold standard, but it takes time, and there is a delay in initiating therapy. Therefore, increased levels of markers may indicate infection, and treatment should be performed accordingly.

The limitations of our study were the small number of patients and the lack of a control group since this was an observational study performed during daily clinical practice. Future prospective studies with many patients will be required to confirm that measurement of neutrophil CD64 expression is a better predictor of local infection than other markers.

## Conclusions

To the best of our knowledge, this is the first study conducted where biomarkers and conventional biomarkers are studied on closed fractures. Most of the studies are on open fractures. The study is going on many patients in our department. We excluded the culture reports since due to the administration of antibiotics, the positivity of the infection could have been reduced. Our future work will be more focused on the pediatric population, and more research is needed for the early diagnosis of the infection. The predicting factors for infection in closed fractures are found to be significantly correlated by the reason by which the injury took place and by the addiction used by the patients rather than due to any underlying disease, and CD64 and CD66 b seem to be promising biomarkers in the diagnosis of early infection. Their elevation at a minimum 10^th^ day after the surgery could be regarded as signs of initiation of the infection rather than waiting for weeks for clinical signs to appear and then starting the treatment for infection. Diagnosing an infection at an early stage could be useful in managing complications that could arise if the infection increases or could not be managed later.
